# Effects of a 6-Week Treadmill Training With and Without Virtual Reality on Frailty in People With Multiple Sclerosis

**DOI:** 10.1016/j.apmr.2024.09.010

**Published:** 2024-09-26

**Authors:** Tobia Zanotto, Irina Galperin, Danya Pradeep Kumar, Anat Mirelman, Shahar Yehezkyahu, Keren Regev, Arnon Karni, Tanja Schmitz-Hübsch, Friedemann Paul, Sharon G. Lynch, Abiodun E. Akinwuntan, Jianghua He, Bruce R. Troen, Hannes Devos, Jeffrey M. Hausdorff, Jacob J. Sosnoff

**Affiliations:** a Department of Occupational Therapy Education, School of Health Professions, University of Kansas Medical Center, Kansas City, KS; b Mobility Core, University of Kansas Center for Community Access, Rehabilitation Research, Education and Service, Kansas City, KS; c Landon Center on Aging, University of Kansas Medical Center, Kansas City, KS; d Center for the Study of Movement, Cognition and Mobility, Neurological Institute, Tel Aviv Sourasky Medical Center, Tel Aviv, Israel; e Department of Neurology, Faculty of Medicine, Tel Aviv University, Tel Aviv, Israel; f Department of Physical Therapy, Rehabilitation Science, and Athletic Training, School of Health Professions, University of Kansas Medical Center, Kansas City, KS; g Sagol School of Neuroscience, Tel Aviv University, Tel Aviv, Israel; h Neuroimmunology and Multiple Sclerosis Unit of the Neurology Division, Tel Aviv Sourasky Medical Center, Tel Aviv, Israel; i Neuroscience Clinical Research Center, Charité – Universitätsmedizin Berlin, Berlin, Germany; j Experimental and Clinical Research Center, Max Delbrück Center for Molecular Medicine and Charité – Universitä;tsmedizin Berlin, Berlin, Germany; k Department of Neurology, Charité – Universitätsmedizin Berlin, Berlin, Germany; l Department of Neurology, School of Medicine, University of Kansas Medical Center, Kansas City, KS; m Department of Biostatistics and Data Science, University of Kansas Medical Center, Kansas City, KS; n Division of Geriatrics, Department of Internal Medicine, School of Medicine, University of Kansas Medical Center, Kansas City, KS; o Research Service, Kansas City Veterans Affairs Healthcare System, Kansas City, MO; p Department of Physical Therapy, Faculty of Medical & Health Sciences, Tel Aviv University, Tel Aviv, Israel; q Rush Alzheimer’ s Disease Center and Department of Orthopaedic Surgery, Rush University Medical Center, Chicago, IL

**Keywords:** Cognitive function, Frailty, Multiple sclerosis, Rehabilitation, Virtual Reality

## Abstract

**Objective:**

To examine the effects of a cognitive-motor rehabilitation program consisting of treadmill training (TT) augmented by virtual reality (TT+VR) on frailty in people with multiple sclerosis (pwMS).

**Design:**

Secondary analysis from a multicenter randomized controlled trial investigating the effects of TT+VR, compared with TT only, on measures of mobility and cognitive function in pwMS.

**Setting:**

Four university research laboratories in 3 countries.

**Participants:**

A total of 124 pwMS were randomized into the parent trial. Here, we studied a subset of n = 83 participants (mean age, 49.4±9.3y; 73.5% female; expanded disability status scale range, 2.0-6.0), who completed the intervention and had complete preintervention and postintervention frailty data.

**Interventions:**

Participants were randomly allocated to TT+VR (n=44) or TT (n=39). Both groups trained 3 times a week for 6 weeks.

**Main Outcome Measures:**

Frailty was assessed using a 40-item frailty index (FI) through standard validated procedures and represented the primary study outcome. Two exploratory frailty indices were also computed by isolating health-related deficits involving the cognitive (FI-physical) or physical (FI-cognitive) domains from the main FI. The assessments were performed at baseline and after 6 weeks, upon intervention completion.

**Results:**

The mean FI of study participants at baseline was 0.33±0.13, indicating a moderate average level of frailty. FI scores improved in both TT+VR and TT groups participants (pooled mean ΔFI, 0.024; 95% CI, 0.010-0.038; F=10.49; *P*=.002; *η*_p_^2^=0.115), without any group-by-time interaction (F=0.82; *P*=.367; *η*_p_^2^=0.010). However, a significant group-by-time interaction was found for pretraining and posttraining changes in FI-cognitive (F=5.74; *P*=.019; *η*_p_^2^=0.066), suggesting a greater improvement for TT+VR group participants than for TT group participants.

**Conclusions:**

TT with or without virtual reality can reduce frailty levels in pwMS. While both TT and TT+VR had a positive effect on overall frailty, only TT+VR improved cognitive aspects of frailty and may represent an appropriate strategy for counteracting frailty in pwMS.

Frailty is a biological syndrome of decreased reserve and resistance to stressors arising from cumulative declines across multiple physiologic systems.^1^ This condition is very common among people living with multiple sclerosis (MS). Indeed, people with MS (pwMS) have a 15-fold higher risk of being frail compared with age-matched individuals living without MS.^2^ Recent studies suggest that up to two-thirds of community-dwelling ambulatory pwMS are frail,^3,4^ and this proportion is even higher (upward of 90%) in patients with more advanced mobility disability.^5^ In addition, frailty within MS is strongly associated with adverse clinical outcomes, such as falls, independent of age, sex, and disability levels.^4^ Consequently, there is a critical need to identify strategies to reduce frailty in pwMS.

In this regard, rehabilitation interventions play a central role in restoring the health and well-being of pwMS.^6^ For instance, physical activity or exercise-based rehabilitation strategies can improve multiple symptoms of MS, including motor^7^ and cognitive dysfunction^8^ and depression,^9^ all of which may be involved in the etiology of frailty in MS.^10^ Rehabilitation strategies involving different types and combinations of physical activity have been shown to reduce frailty levels, or mitigate hallmarks of frailty, in community-dwelling older adults.^11,12^ This provides a strong rationale for the potential effectiveness of physical activity interventions to minimize frailty in pwMS. To date, however, it is still not clear if rehabilitation interventions can reduce frailty in this clinical population. The early onset of frailty in individuals with MS^2^ further underscores the critical importance of timely interventions to manage this condition.

Since cognitive and motor dysfunction are the most common symptoms of MS,^13,14^ multimodal cognitive-motor rehabilitation has been gaining popularity in recent years as a strategy to promote overall well-being in pwMS.^15–17^ Importantly, a recent randomized controlled trial (RCT) has shown promising results concerning the effectiveness of a cognitive-motor intervention (stationary cycling through a virtual environment) in reducing frailty among older adults living in a long-term care setting.^18^ In addition, our recently completed RCT focusing on virtual reality (VR) treadmill training (TT) demonstrated significant improvements in hallmarks of frailty, such as walking, cognition, and depression, in pwMS.^19^ Importantly, the TT augmented by VR (VR+TT) used in our previous investigation was originally conceived to target both gait and cognitive issues,^20^ which are both implicated in the etiology of frailty,^21,22^ and to reduce the risk of falling, a significant corollary measure of frailty.^20^ However, it is currently unknown whether multimodal cognitive- motor rehabilitation can reduce frailty levels in pwMS.

The purpose of this study was to address the critical knowledge gap concerning whether frailty is modifiable in pwMS and whether cognitive-motor rehabilitation produces a stronger effect on frailty than motor rehabilitation alone. Specifically, our objective was to examine the effects of VR-based augmentation of TT on frailty in pwMS. We hypothesized that participants randomized to TT+VR would exhibit a greater reduction in frailty compared with participants randomized to TT only.

## Methods

### Study design and setting

The current study is a secondary analysis of the Virtual Reality for MS (VR4MS) study, a multicenter RCT with a 2-arm, single-blind design that took place at 4 clinical sites in Israel (Tel Aviv Sourasky Medical Center), in the United States (University of Kansas Medical Center and University of Illinois at Urbana-Champaign), and Germany (NeuroCure Clinical Research Center, Charité-Universitütsmedizin in Berlin). This RCT was conducted between August 2016 and June 2021, and the main study findings were published.^19^ Here, we set out to examine the effects of the VR4MS intervention on frailty data that were collected as part of the trial.

### Study participants

Participants were pwMS who completed the baseline and post-training assessments of the VR4MS trial. Briefly, the inclusion criteria were as follows: (1) between 20 and 65 years of age; (2) diagnosis of relapsing-remitting MS confirmed by a physician; (3) score between 2.0 and 6.0 on the Expanded Disability Status Scale; (4) MS-related gait limitations (based on a score ≥2 in the first question of the MS Walking Scale^23^). Participants who met any of the following criteria were excluded: (1) unable to walk unassisted for 5 minutes; (2) diagnosis of another neurologic disorder; (3) a score <24 on the Mini-Mental Status Examination; (4) diagnosis of an active psychiatric condition (eg, severe depression); (5) any orthopedic, cardiovascular, or other problems that may interfere with the ability to walk. All participants provided written informed consent prior to taking part in the study. The study conformed to the ethical standards for medical research involving human subjects, as laid out in the Declaration of Helsinki. All procedures were reviewed and approved by the local institutional review boards of the participating centers.

### Procedures

All study procedures pertaining to the intervention have been detailed in the study protocol^24^ and the main manuscript.^19^ Here, we provide a summary of the main aspects. Briefly, participants were randomized to either 6 weeks of TT alone (ie, the active-control group) or 6 weeks of TT+VR (ie, the experimental group), after the baseline assessment. The randomization sequence was generated in MATLAB^a^ and exclusively shared with the trainers on a rolling, as-needed basis to conceal the allocation. All study assessors were blinded to group allocation. Participants in both groups received at least 15 and up to 18 training sessions (thrice weekly for 6wk). Participants in both groups wore a safety harness during training. As part of TT+VR, participants walked on the treadmill while navigating a virtual environment projected on a television screen by means of a VR system (GaitBetter^b^). The VR system provided additional motor and cognitive challenges, including obstacle crossing, planning, and divided attention tasks individualized to the participant’s level of performance. The TT speed for both groups was selected based on the participant’s over-ground walking speed, measured during the baseline assessment, and progressed throughout the 6-week training period according to the protocol.^24^ After the 6-week intervention, all participants returned to the local study center (within 10d) to complete the posttraining assessment procedures, which were identical to the baseline assessment.

### Outcome measures

For this secondary analysis, the main study outcome was frailty, evaluated through a frailty index (FI) based on the deficit accumulation model.^25,26^ The FI was calculated from 40 deficits in a wide range of health domains (ie, global health; physical, cognitive, and psychosocial function; comorbidities). The individual health-related deficits were identified through a combination of objective and self-reported measures, collected as part of the baseline and posttraining assessments, and in agreement with the guiding principles of the deficit accumulation model.^26^ Specifically, the objective/self-reported measures were extracted from the following: (1) the 6-minute walk test; (2) the 25-foot walk test; (3) the 54-item MS quality of life questionnaire^27^; (4) the modified fatigue impact scale; (5) the international physical activity questionnaire^28^; (6) the Symbol Digit Modalities Test; (7) the California Verbal Learning Test; (8) the Trail Making Test^29^; (9) the Brief Visuospatial Learning Test^30^; (10) a standardized health survey. The full operationalization of the FI is summarized in Supplemental table S1 (available online only at http://www.archives-pmr.org/). FI scores ≤0.24 were used to indicate a nonfrail state, while FI scores between 0.24 and 0.36 (ie, >0.24; ≤0.36) and >0.36 were used to indicate moderate and severe frailty, respectively.^31^ The number of frailty transitions (eg, severe to moderate frailty or vice versa) after the 6-week intervention was recorded. In addition, we classified participants as nonresponders if they had a ΔFI score (reduction) <0.028^32^ and as moderate or large responders if they had a ΔFI score (reduction) between 0.028 and 0.076 or >0.076, respectively.^32,33^

For exploratory analysis purposes, we then computed two additional frailty indices to examine the potentially distinct effects of the experimental intervention on the physical and cognitive aspects of frailty in the study population. In the first index, we removed all health-related deficits pertaining to the cognitive domain from the operational definition, and we named this measure “FI-physical” (ie, a FI emphasizing the physical aspects of frailty). Conversely, in the second index, we removed all health-related deficits pertaining to the physical domain from the operational definition, and we named this measure “FI-cognitive” (ie, a FI emphasizing the cognitive aspects of frailty). The exact operationalizations of FI-physical and FI-cognitive are fully described in Supplemental tables S2 and S3 (available online only at http://www.archives-pmr.org/).

### Statistical analysis

Statistical analyses were performed in SPSS, version 29.0.^c^ The Kolmogorov-Smirnov test was used to determine whether the data were normally distributed. Differences in baseline demographic characteristics and clinical characteristics between participants randomized to TT and participants randomized to TT+VR were explored through independent *t* tests or Mann-Whitney *U* tests, as appropriate (continuous variables), or by means of Chi-square tests (categorical variables). To examine the differences in FI before and after the intervention in the 2 groups (TT and TT+VR), mixed (2 × 2 design) analysis of variance (ANOVA) tests were conducted. The Levene’s test was used to check the homogeneity of variance assumption. For the purposes of this secondary analysis, we used a “per protocol” analysis (ie, complete case analysis method) to report the main findings. However, a modified intention-to-treat analysis was also conducted as a sensitivity analysis to examine the confounding effect of dropouts. To this end, we utilized an imputation approach consisting of replacing missing posttraining FI values with a participant’s baseline FI values. Analogously, 2 additional mixed-design ANOVA tests were performed, as exploratory analyses, to analyze the effects of TT and TT+VR on FI-physical and FI-cognitive. In addition, we examined the concurrent validity of the exploratory outcomes, FI-physical and FI-cognitive, with respect to overall frailty (ie, FI) through Pearson’s correlations. Furthermore, to evaluate the criterion validity of the 2 sensitivity FI measures, we explored the association between FI-physical/FI-cognitive and the number of falls reported by participants in the previous 12 months (at baseline) by means of negative binomial regression analyses. Statistical limits for interpretation of the analyses were set at *P*<.05.

## Results

### Study participants

**Fig 1:**
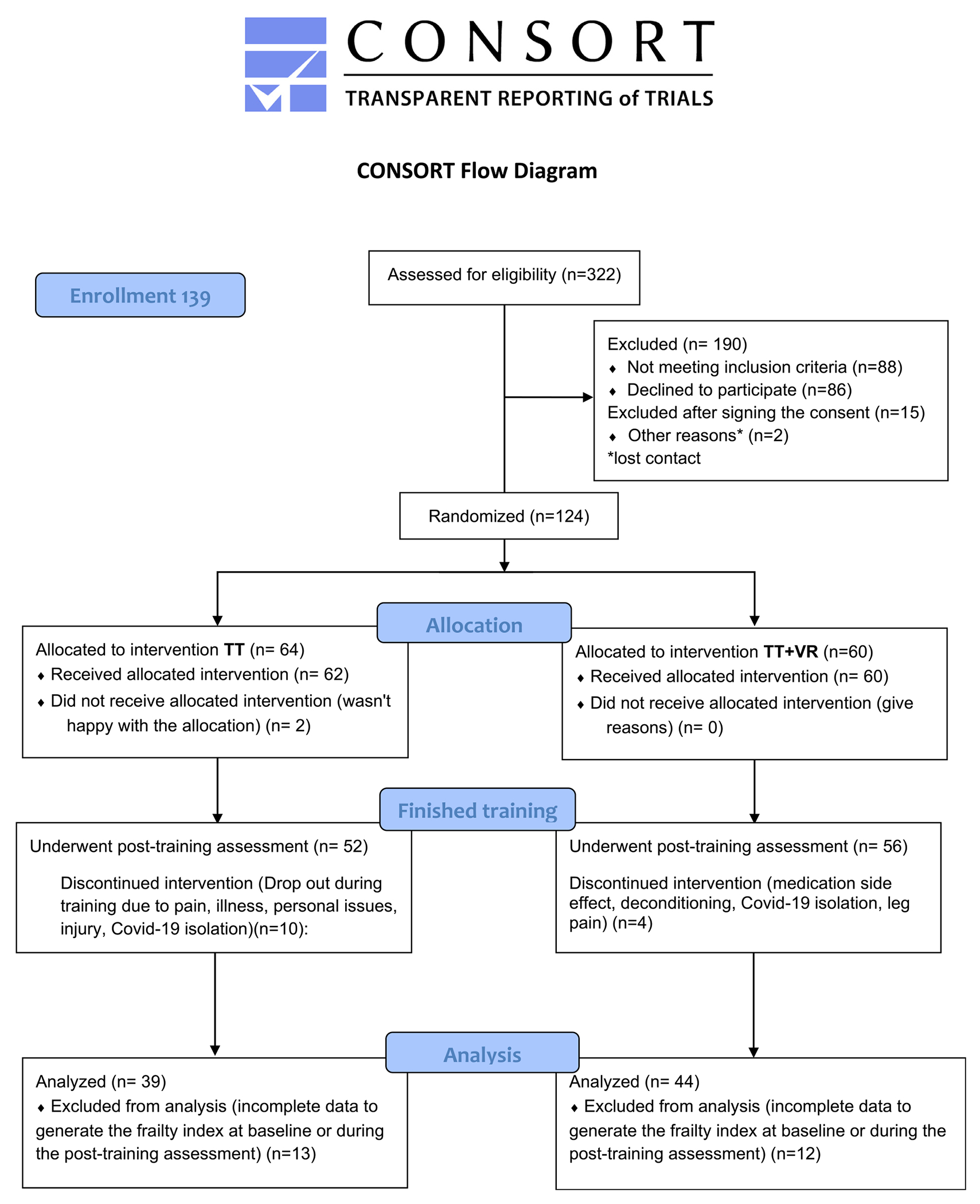
Modified Consolidated Standards of Reporting Trials (CONSORT) flowchart.

Overall, 139 participants were enrolled in the parent trial, of whom 124 completed the baseline assessment and were randomly allocated to TT (n=64) or TT+VR (n=60). While 108 participants completed the training program, 25 were excluded from this analysis because they had more than 10 missing items on the FI (either at the baseline or posttraining assessment), as recommended by best-practice guidelines.^26^ Therefore, 83 participants (39 and 44 in the TT and TT+VR groups, respectively) were included in this analysis. The participant flow, including reasons for exclusion at different study stages, is fully summarized in the modified Consolidated Standards of Reporting Trials diagram (fig 1). The baseline demographic and clinical characteristics of the study participants are summarized in table 1. No differences between participants randomized to TT and TT+VR were found, indicating that the 2 groups were well-matched in terms of demographic characteristics, frailty, and other clinical characteristics.

### Training effects on frailty

**Fig 2:**
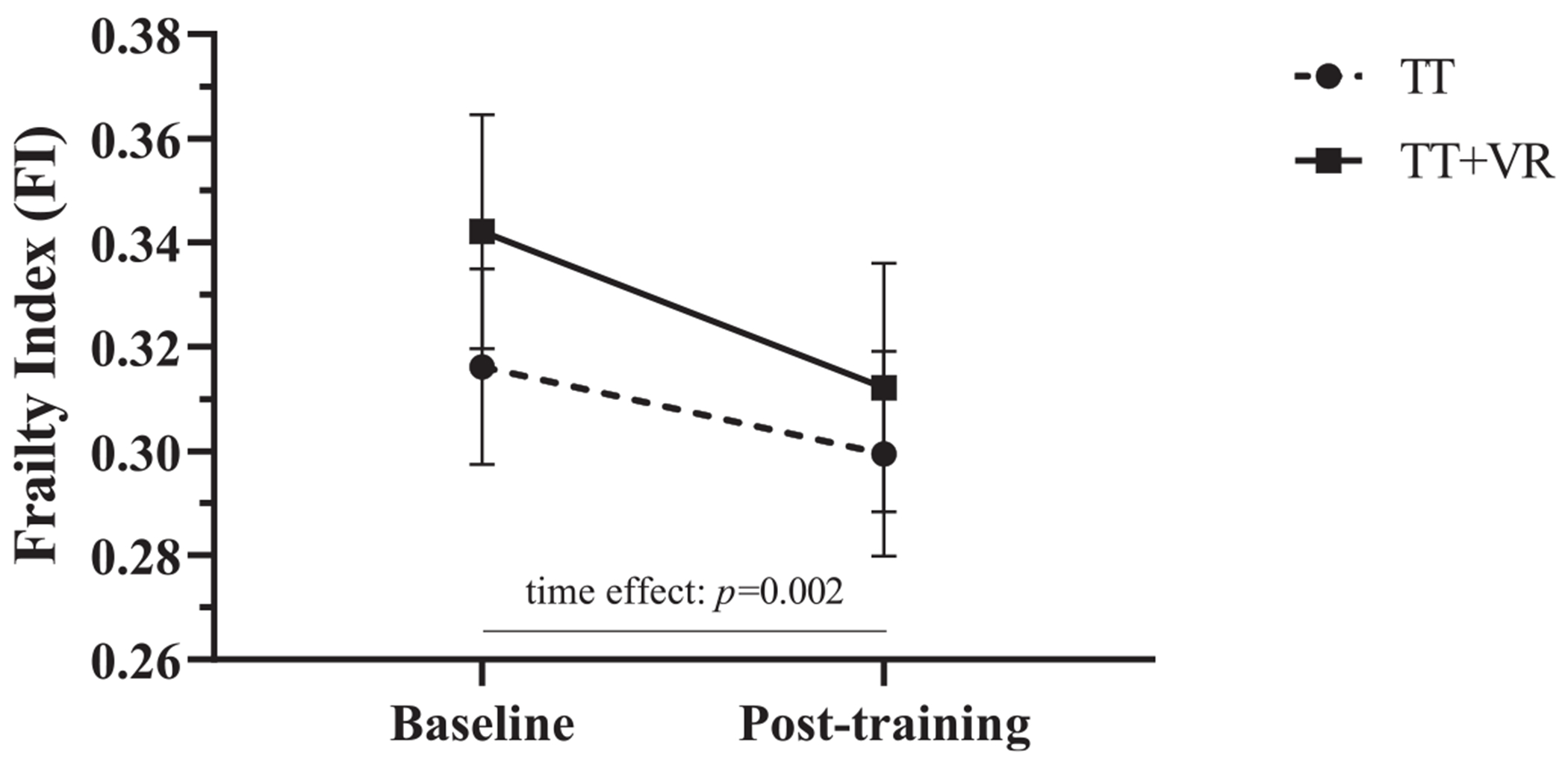
Mean FI scores and SEM at baseline and after 6 weeks (after training).

**Fig 3:**
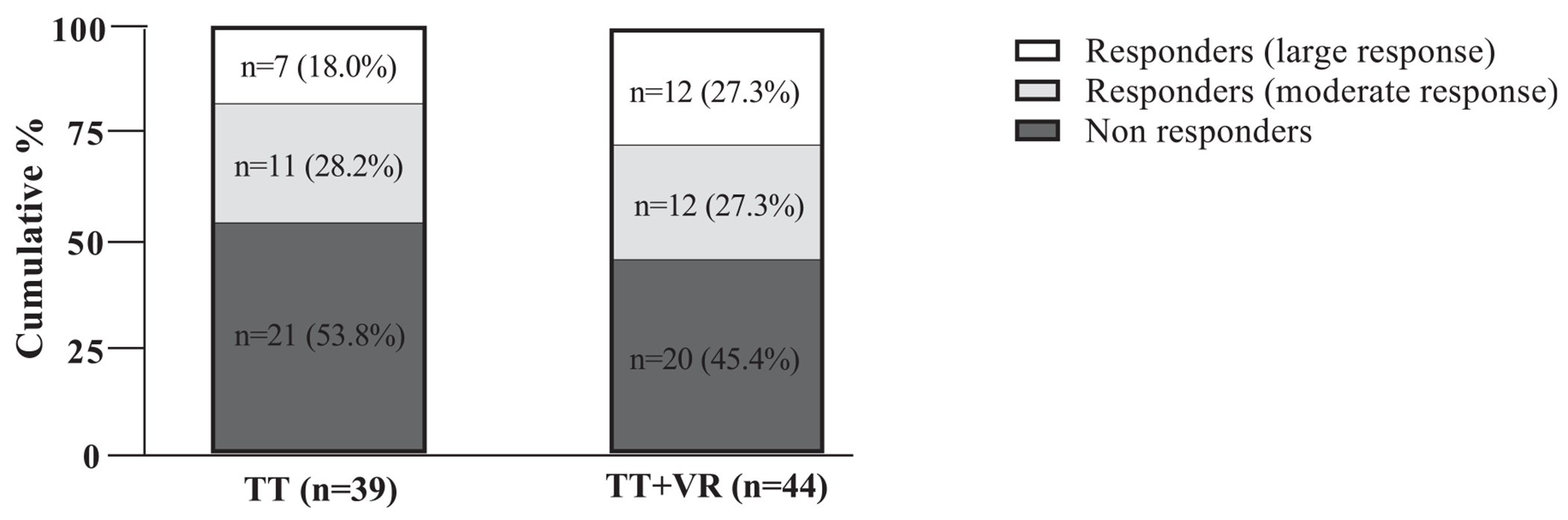
Proportion of responders and nonresponders in the 2 study groups. Nonresponders: ΔFI score <0.028; responders (moderate response): 0.028≤ΔFI score<0.076; responders (large response): ΔFI score >0.076.

Figure 2shows the changes in frailty levels in both groups after 6 weeks of training. The per-protocol mixed-design ANOVA revealed that FI scores became lower (indicating a reduction in frailty) in both TT and TT+VR after the intervention (mean ΔFI, 0.024; 95% CI, 0.010-0.038; time effect: F=10.49; *P*=.002; *η*_p_^2^=0.115). No significant group-by-time interactions were found for this analysis (F=0.82; *P*=.367; *η*_p_^2^=0.010). The mean ΔFI for TT and TT+VR participants was 0.017 (95% CI, 0.005-0.038) and 0.030 (95% CI, 0.010-0.050), respectively. The sensitivity-modified intention-to-treat analysis yielded similar findings, namely, a significant time effect (F=11.00; *P*=.001; *η*_p_^2^=0.104) but no group-by-time interactions (F=1.44; *P*=.233; *η*_p_^2^=0.015). Overall, 42 (50.6%) and 41 (49.4%) study participants were classified as responders and nonresponders, respectively, based on a clinically meaningful change in FI >0.028.^32^ Figure 3 shows the proportion of responders and nonresponders to the intervention as a function of the intervention arm. Additionally, among the 42 responders, 17 (20.5%) individuals also exhibited a change in frailty status. Specifically, 11 (13.3%) participants transitioned from severe frailty to moderate frailty, while 6 (7.2%) participants transitioned from moderate frailty to not frail. Among the 41 nonresponders, 5 (6.0%) participants had worsened frailty status after the intervention.

**Fig 4:**
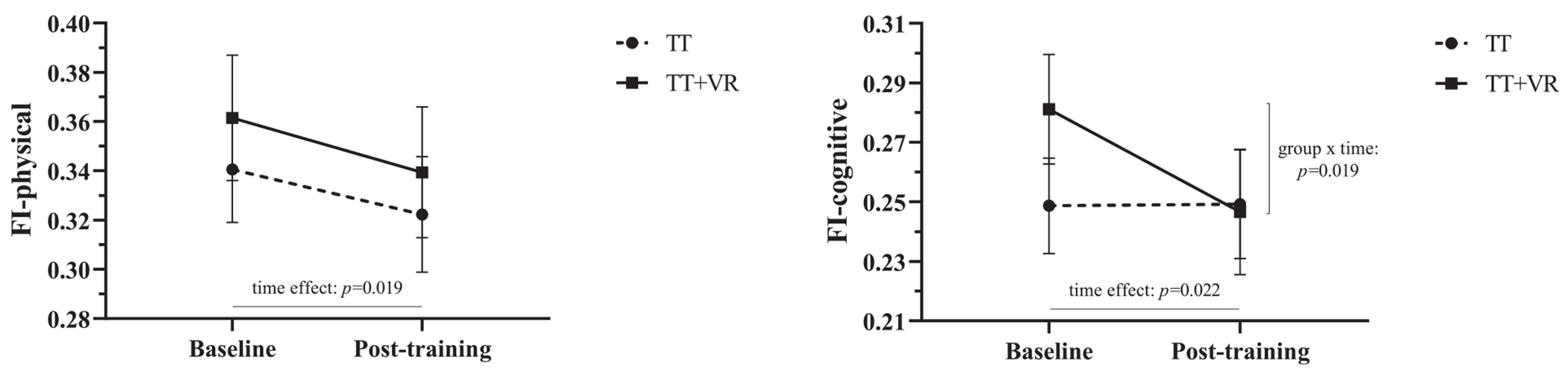
Mean FI-physical and FI-cognitive scores and SEM at baseline and after 6 weeks (after training).

The exploratory analyses confirmed a significant effect of time on both FI-physical (F=5.75; *P*=.019; *η*_p_^2^=0.066) and FI-cognitive (F=5.43; *P*=.022; *η*_p_^2^=0.063). A significant group-by-time interaction was found for FI-cognitive (F=5.74; *P*=.019; *η*_p_^2^=0.066) but not for FI-physical (F=0.52; *P*=.820; *η*_p_^2^=0.001). Figure 4 depicts changes in FI-physical and FI-cognitive as a function of the intervention arm.

Both FI-physical (*r*=0.970; *P*<.001) and FI-cognitive (*r*=0.846; *P*<.001) exhibited concurrent validity, as they were strongly correlated with FI scores. In addition, the negative binomial regression analyses revealed that both FI-physical (incidence rate ratio, 2.73; 95% CI, 1.39-5.39; *P*=.004) and FI-cognitive (incidence rate ratio, 2.25; 95% CI, 1.20-4.19; *P*=.011) were associated with a higher number of falls reported at baseline by the study participants.

## Discussion

In this study, we aimed to provide evidence that a rehabilitation intervention can reduce frailty levels in pwMS. Both TT and TT+VR resulted in reduced FI scores after 6 weeks of training in the study participants. Our hypothesis that cognitive-motor rehabilitation (ie, TT+VR) would reduce frailty to a greater extent than TT alone was not confirmed. However, participants who were randomized to TT+VR exhibited a greater benefit on FI-cognitive than participants who underwent TT. This observation seems to suggest that, compared with physical training alone, cognitive-motor rehabilitation interventions may represent a more appropriate approach to counteracting the cognitive aspects of frailty in pwMS.

Minimizing frailty in the MS community is clinically important, as frailty increases the vulnerability to adverse outcomes that are very common among pwMS, such as falls,^4^ and may aggravate signs and symptoms of MS.^34^ In addition, owing to the combined effects of premature frailty and progressive aging of pwMS, timely management of frailty will likely represent an increasing challenge for the long-term care of MS populations. The current investigation provides the first evidence that a rehabilitation intervention consisting of treadmill walking, with or without VR, may be a viable strategy to minimize frailty levels in ambulatory pwMS. Interestingly, previous research conducted in communitydwelling older adults concluded that changes in FI scores >0.028 and >0.030 (expressed as absolute values) represent clinically meaningful changes in frailty.^32,33^ While some methodological considerations limit direct comparisons with these investigations, it is interesting to note that the change in FI score exhibited by TT+VR participants in the current analysis is compatible with a clinically meaningful change in frailty (fig 2).^33^ In addition, analogous to these observations, participants who were randomized to TT+VR also had a slightly higher proportion of responders to the intervention than those randomized to TT (fig 3), which was more noticeable for the proportion of individuals who had a large response (27.3% vs 18.0%). However, regardless of the training modality, it should be highlighted that a relatively high proportion of participants (20.5%) exhibited a transition (ie, improvement) in frailty status. This underscores the critical importance of rehabilitation, not only to reduce but also to potentially reverse frailty in MS. Relatedly, we also note that these frailty transitions were observed after only 6 weeks of training. Because longer training durations (eg, 5mo) have been linked to greater improvements in frailty outcomes in geriatric populations,^35^ it is possible that prolonging TT and TT+VR beyond 6 weeks would have resulted in greater benefits in terms of frailty reduction and the number of observed transitions in frailty status.

Interestingly, the exploratory analyses (fig 4) revealed that TT and TT+VR had a similar and beneficial effect on FI-physical, but only TT+VR reduced FI-cognitive. This seems to suggest that both training modalities were effective in positively modifying frailty aspects related to physical function. On the other hand, only cognitive-motor rehabilitation (ie, TT+VR) led to improvements in frailty aspects that were not related to physical function and encompassing cognition. This finding may have important rehabilitation implications, as cognitive dysfunction is one of the most common symptoms in pwMS and could be one of the underlying factors of MS-related frailty.^36^ In this respect, a multimodal rehabilitation intervention capable of targeting both physical and cognitive aspects of frailty may be more advantageous than an intervention focusing exclusively on physical rehabilitation. Another advantage of cognitive-motor rehabilitation is that this type of intervention modality is often perceived as more engaging than physical rehabilitation alone.^37^ For instance, in their recent study involving older adults living with dementia, Karssemeijer et al^18^ reported a trend toward higher adherence in participants who underwent stationary cycling enhanced by VR compared with participants who performed stationary cycling only (87.3% vs 81.1%; *P*=.05). These findings are mirrored by the lower dropout rate observed in TT+VR participants, compared with TT participants, in our study (6.7% vs 18.8%; fig 1).

### Study limitations

Several strengths and limitations of the current investigation should be acknowledged. First of all, this is the first study providing any evidence that frailty is modifiable in pwMS. Second, by choosing the FI as the main study outcome, we sought to address a common methodological limitation that has plagued the field. Indeed, a recent review has highlighted the critical need for studies that include a frailty outcome, rather than exclusively relying on hallmarks of frailty, to better gauge the potential of preventive and rehabilitative interventions on frailty.^38^ On the other hand, the secondary research nature of our investigation represents a study limitation. For instance, the lack of a passive (or sham treatment) control group should be acknowledged as a limitation. Relatedly, this secondary analysis included only a subset of participants from the parent trial, and the sample size calculation was exclusively performed for the original study.^19,24^ In addition, FI-physical and FI-cognitive should be considered exploratory outcomes, and the effects of the intervention on these measures of frailty warrant some caution. For instance, although we used best practice guidelines in designing these outcomes,^26^ the content validity of such measures has not been established yet.

## Conclusions

This study provides evidence that a 6-week rehabilitation intervention consisting of TT, augmented or not by VR, reduces frailty levels in pwMS. While both TT and TT+VR had a positive effect on overall frailty, only TT+VR participants exhibited benefits in cognitive aspects of frailty at the end of the intervention. While this study provided the first evidence that frailty is modifiable in pwMS, further research is needed. Future research efforts should also be directed toward addressing the frailty rehabilitation needs of nonambulatory individuals living with MS.

## Supplementary Material

Supplement

## Figures and Tables

**Table 1:** Baseline participant characteristics

Variables	All Participants (N=83)	TT (n=39)	TT + VR (n=44)	*P* Value
Female sex (%)	61 (73.5)	28 (71.8)	33 (75.0)	.741
Age (y)	49.4±9.3	49.2±9.5	49.5±9.2	.896
Body mass (kg)	70.8 (25.1)	70.1 (24.0)	70.8 (28.5)	.695
Height (cm)	168.2±9.5	166.5±9.1	169.8±9.7	.111
BMI (kg/m^2^)	25.0 (8.3)	25.2 (9.8)	24.9 (8.7)	.622
Education (y)	16.0 (3.0)	16.0 (2.0)	16.0 (5.0)	.560
EDSS (score)	3.5 (2.0)	3.5 (3.5)	3.5 (1.5)	.380
FI (score)	0.33±0.13	0.32±0.18	0.34±0.15	.386
FI-physical (score)	0.35±0.15	0.34±0.13	0.36±0.17	.535
FI-cognitive (score)	0.27±0.11	0.25±0.10	0.28±0.12	.194

Results are expressed as frequencies and percentages for categorical variables and mean ± SD or median (interquartile range) for continuous variables. Abbreviations: BMI: body mass index; EDSS: expanded disability status scale.

## List of abbreviations:

ANOVA: analysis of variance
FI: frailty index
MS: multiple sclerosis
pwMS: people with multiple sclerosis
RCT: randomized controlled trial
TT: treadmill training
TT+VR: treadmill training augmented by virtual reality
VR: virtual reality
