# Supplementary material for: Effects of a 6-Week Treadmill Training With and Without Virtual Reality on Frailty in People With Multiple Sclerosis
Source: Arch Phys Med Rehabil. Author manuscript; Available in PMC 2026 Apr 20. (PMC13095348; doi:10.1016/j.apmr.2024.09.010)
Supplement: Supplement [file NIHMS2162988-supplement-Supplement.docx]

**Table S1.** Operational definition of the frailty index (FI).

| **Items** | **Source** | **Domain/subdomain** | **Coding** |
| --- | --- | --- | --- |
| 1. Self-rated health? | MSQOL-54 | Global health | Poor = 1; Fair = 0.75; Good = 0.5; Very good = 0.25; Excellent = 0 |
| 2. How has your health changed in the last year? | MSQOL-54 | Global health | Much worse = 1; Somewhat worse = 0.5; Better/Same = 0 |
| 3. Are you using a walking aid? | Patient characteristics | Physical/function in ADLs | Yes = 1; No = 0 |
| 4. Cut down on usual activity in the past month? | MSQOL-54 | Physical/function in ADLs | Yes = 1; No = 0 |
| 5. Does your health limit you in bathing/dressing? | MSQOL-54 | Physical/function in ADLs | Yes = 1; A little = 0.5; No = 0 |
| 6. Does your health limit you in lifting/carrying groceries? | MSQOL-54 | Physical/function in ADLs | Yes = 1; A little = 0.5; No = 0 |
| 7. Does your health limit you in climbing one flight of stairs? | MSQOL-54 | Physical/function in ADLs | Yes = 1; A little = 0.5; No = 0 |
| 8. Does your health limit you in walking one block? | MSQOL-54 | Physical/function in ADLs | Yes = 1; A little = 0.5; No = 0 |
| 9. Does your health limit you in bending, kneeling or stooping? | MSQOL-54 | Physical/function in ADLs | Yes = 1; A little = 0.5; No = 0 |
| 10. Moderate activity in leisure time in the past week? | IPAQ | Physical/activity levels | Yes = 0; No = 1 |
| 11. Walking in leisure time in the past week? | IPAQ | Physical/activity levels | Yes = 0; No = 1 |
| 12. How many hours did you spend sitting during a weekend day in the past week? | IPAQ | Physical/activity levels | ≥6 hours = 1; <6 hours = 0 |
| 13. 6MWT performance | 6MWT | Physical/performance | 6MWD <350 m = 1; 6MWD ≥ 350 m = 0 |
| 14. 25-foot walk performance | 25-foot walk test | Physical/performance | <1 m/s = 1; ≥1 m/s = 0 |
| 15. Did you feel tired in the past month? | MSQOL-54 | Physical/energy | All of the time = 1; Most of the time = 0.8; A good bit of the time = 0.6; Some of the time = 0.4; A little = 0.2; None of the time = 0 |
| 16. Did you have a lot of energy in the past month? | MSQOL-54 | Physical/energy | All of the time = 0; Most of the time = 0.2; A good bit of the time = 0.4; Some of the time = 0.6; A little = 0.8; None of the time = 1 |
| 17. Muscle weakness due to fatigue in the past month? | MFIS | Physical/energy | Always = 1; Often = 0.75; Sometimes = 0.5; Rarely = 0.25; Never = 0 |
| 18. Need to rest more often due to fatigue in the past month? | MFIS | Physical/energy | Always = 1; Often = 0.75; Sometimes = 0.5; Rarely = 0.25; Never = 0 |
| 19. MMSE score | MMSE | Cognitive/global | <20 = 1; 20–24 = 0.5; >24 = 0 |
| 20. CVLT-II | CVLT-II | Cognitive/verbal learning | Total words recalled: above first quintile of distribution = 0; lowest quintile = 1 |
| 21. BVMT | BVMT | Cognitive/visuospatial | Total BVMT score: above first quintile of distribution = 0; lowest quintile = 1 |
| 22. TMT | TMT | Cognitive/working memory | TMT-A time ≤ 78s = 0; > 78s = 1 |
| 23. SDMT | SDMT | Cognitive/information processing | Raw SDMT score: above first quintile of distribution = 0; lowest quintile = 1 |
| 24. Have you had troubles with memory in the past month? | MSQOL-54 | Cognitive/general memory | All of the time = 1; Most of the time = 0.8; A good bit of the time = 0.6; Some of the time = 0.4; A little = 0.2; None of the time = 0 |
| 25. Difficulty concentrating or thinking in the past month? | MSQOL-54 | Cognitive/attentional control | All of the time = 1; Most of the time = 0.8; A good bit of the time = 0.6; Some of the time = 0.4; A little = 0.2; None of the time = 0 |
| 26. Been less motivated to participate in social activities in the past month? | MFIS | Psychosocial/participation | Always = 1; Often = 0.75; Sometimes = 0.5; Rarely = 0.25; Never = 0 |
| 27. Have you been happy in the past month? | MSQOL-54 | Psychosocial/mood | All of the time = 0; Most of the time = 0.2; A good bit of the time = 0.4; Some of the time = 0.6; A little = 0.8; None of the time = 1 |
| 28. Have you felt downhearted and blue in the past month? | MSQOL-54 | Psychosocial/mood | All of the time = 1; Most of the time = 0.8; A good bit of the time = 0.6; Some of the time = 0.4; A little = 0.2; None of the time = 0 |
| 29. Accomplished less than you would like as a result of emotional problems in the past month? | MSQOL-54 | Psychosocial/mood | Yes = 1; No = 0 |
| 30. Do you take more than 3 medications per day? | Medication list | Comorbidity/polypharmacy | Yes = 1, No = 0 |
| 31. Body mass index | Patient characteristics | Comorbidity/body weight | <18.5, ≥30 = 1; >25, <30 = 0.5; ≥18.5, ≤25 = 0 |
| 32. How much bodily pain have you had in the past month? | MSQOL-54 | Comorbidity/pain | Very severe = 1; Severe = 0.8; Moderate = 0.6; Mild = 0.4; Very mild = 0.2; None = 0 |
| 33. Satisfied with sexual function in the past month? | MSQOL-54 | Comorbidity/sexual | Very dissatisfied = 1; Somewhat dissatisfied = 0.5; Neither dissatisfied nor satisfied = 0 |
| 34. Difficulty getting/keeping erection (male) or inadequate lubrication (female) in the past month? | MSQOL-54 | Comorbidity/sexual | Very much a problem = 1; Somewhat of a problem = 0.5; Little of a problem = 0 |
| 35. Cardiovascular disease | Patient characteristics | Comorbidity/disease | Yes = 1; Suspected = 0.5; No = 0 |
| 36. Stroke | Patient characteristics | Comorbidity/disease | Yes = 1; Suspected = 0.5; No = 0 |
| 37. Diabetes | Patient characteristics | Comorbidity/disease | Yes = 1; Suspected = 0.5; No = 0 |
| 38. Cancer | Patient characteristics | Comorbidity/disease | Yes = 1; Suspected = 0.5; No = 0 |
| 39. Arthritis | Patient characteristics | Comorbidity/disease | Yes = 1; Suspected = 0.5; No = 0 |
| 40. Chronic lung disease | Patient characteristics | Comorbidity/disease | Yes = 1; Suspected = 0.5; No = 0 |

**Abbreviations:** MSQOL-54: 54-item multiple sclerosis quality of life questionnaire; MFIS: modified fatigue impact scale; IPAQ: international physical activity questionnaire; MMSE: mini mental status examination; CVLT-II: California verbal learning test; BVMT: brief visuospatial memory test; TMT: trail making test; SDMT: symbol digit modalities test; 6MWT: six-minute walk test.

**Table S2.** Operational definition of FI-physical.

| **Items** | **Source** | **Domain/subdomain** | **Coding** |
| --- | --- | --- | --- |
| 1. Self-rated health? | MSQOL-54 | Global health | Poor = 1; Fair = 0.75; Good = 0.5; Very good = 0.25; Excellent = 0 |
| 2. How has your health changed in the last year? | MSQOL-54 | Global health | Much worse = 1; Somewhat worse = 0.5; Better/Same = 0 |
| 3. Are you using a walking aid? | Patient characteristics | Physical/function in ADLs | Yes = 1; No = 0 |
| 4. Cut down on usual activity in the past month? | MSQOL-54 | Physical/function in ADLs | Yes = 1; No = 0 |
| 5. Does your health limit you in bathing/dressing? | MSQOL-54 | Physical/function in ADLs | Yes = 1; A little = 0.5; No = 0 |
| 6. Does your health limit you in lifting/carrying groceries? | MSQOL-54 | Physical/function in ADLs | Yes = 1; A little = 0.5; No = 0 |
| 7. Does your health limit you in climbing one flight of stairs? | MSQOL-54 | Physical/function in ADLs | Yes = 1; A little = 0.5; No = 0 |
| 8. Does your health limit you in walking one block? | MSQOL-54 | Physical/function in ADLs | Yes = 1; A little = 0.5; No = 0 |
| 9. Does your health limit you in bending, kneeling or stooping? | MSQOL-54 | Physical/function in ADLs | Yes = 1; A little = 0.5; No = 0 |
| 10. Moderate activity in leisure time in the past week? | IPAQ | Physical/activity levels | Yes = 0; No = 1 |
| 11. Walking in leisure time in the past week? | IPAQ | Physical/activity levels | Yes = 0; No = 1 |
| 12. How many hours did you spend sitting during a weekend day in the past week? | IPAQ | Physical/activity levels | ≥6 hours = 1; <6 hours = 0 |
| 13. 6MWT performance | 6MWT | Physical/performance | 6MWD <350 m = 1; 6MWD ≥ 350 m = 0 |
| 14. 25-foot walk performance | 25-foot walk test | Physical/performance | <1 m/s = 1; ≥1 m/s = 0 |
| 15. Did you feel tired in the past month? | MSQOL-54 | Physical/energy | All of the time = 1; Most of the time = 0.8; A good bit of the time = 0.6; Some of the time = 0.4; A little = 0.2; None of the time = 0 |
| 16. Did you have a lot of energy in the past month? | MSQOL-54 | Physical/energy | All of the time = 0; Most of the time = 0.2; A good bit of the time = 0.4; Some of the time = 0.6; A little = 0.8; None of the time = 1 |
| 17. Muscle weakness due to fatigue in the past month? | MFIS | Physical/energy | Always = 1; Often = 0.75; Sometimes = 0.5; Rarely = 0.25; Never = 0 |
| 18. Need to rest more often due to fatigue in the past month? | MFIS | Physical/energy | Always = 1; Often = 0.75; Sometimes = 0.5; Rarely = 0.25; Never = 0 |
| 19. Been less motivated to participate in social activities in the past month? | MFIS | Psychosocial/participation | Always = 1; Often = 0.75; Sometimes = 0.5; Rarely = 0.25; Never = 0 |
| 20. Have you been happy in the past month? | MSQOL-54 | Psychosocial/mood | All of the time = 0; Most of the time = 0.2; A good bit of the time = 0.4; Some of the time = 0.6; A little = 0.8; None of the time = 1 |
| 21. Have you felt downhearted and blue in the past month? | MSQOL-54 | Psychosocial/mood | All of the time = 1; Most of the time = 0.8; A good bit of the time = 0.6; Some of the time = 0.4; A little = 0.2; None of the time = 0 |
| 22. Accomplished less than you would like as a result of emotional problems in the past month? | MSQOL-54 | Psychosocial/mood | Yes = 1; No = 0 |
| 23. Do you take more than 3 medications per day? | Medication list | Comorbidity/polypharmacy | Yes = 1, No = 0 |
| 24. Body mass index | Patient characteristics | Comorbidity/body weight | <18.5, ≥30 = 1; >25, <30 = 0.5; ≥18.5, ≤25 = 0 |
| 25. How much bodily pain have you had in the past month? | MSQOL-54 | Comorbidity/pain | Very severe = 1; Severe = 0.8; Moderate = 0.6; Mild = 0.4; Very mild = 0.2; None = 0 |
| 26. Satisfied with sexual function in the past month? | MSQOL-54 | Comorbidity/sexual | Very dissatisfied = 1; Somewhat dissatisfied = 0.5; Neither dissatisfied nor satisfied = 0 |
| 27. Difficulty getting/keeping erection (male) or inadequate lubrication (female) in the past month? | MSQOL-54 | Comorbidity/sexual | Very much a problem = 1; Somewhat of a problem = 0.5; Little of a problem = 0 |
| 28. Cardiovascular disease | Patient characteristics | Comorbidity/disease | Yes = 1; Suspected = 0.5; No = 0 |
| 29. Stroke | Patient characteristics | Comorbidity/disease | Yes = 1; Suspected = 0.5; No = 0 |
| 30. Diabetes | Patient characteristics | Comorbidity/disease | Yes = 1; Suspected = 0.5; No = 0 |
| 31. Cancer | Patient characteristics | Comorbidity/disease | Yes = 1; Suspected = 0.5; No = 0 |
| 32. Arthritis | Patient characteristics | Comorbidity/disease | Yes = 1; Suspected = 0.5; No = 0 |
| 33. Chronic lung disease | Patient characteristics | Comorbidity/disease | Yes = 1; Suspected = 0.5; No = 0 |

**Abbreviations:** MSQOL-54: 54-item multiple sclerosis quality of life questionnaire; MFIS: modified fatigue impact scale; IPAQ: international physical activity questionnaire; 6MWT: six-minute walk test.

**Table S3.** Operational definition of FI-cognitive.

| **Items** | **Source** | **Domain/subdomain** | **Coding** |
| --- | --- | --- | --- |
| 1. Self-rated health? | MSQOL-54 | Global health | Poor = 1; Fair = 0.75; Good = 0.5; Very good = 0.25; Excellent = 0 |
| 2. How has your health changed in the last year? | MSQOL-54 | Global health | Much worse = 1; Somewhat worse = 0.5; Better/Same = 0 |
| 3. MMSE score | MMSE | Cognitive/global | <20 = 1; 20–24 = 0.5; >24 = 0 |
| 4. CVLT-II | CVLT-II | Cognitive/verbal learning | Total words recalled: above first quintile of distribution = 0; lowest quintile = 1 |
| 5. BVMT | BVMT | Cognitive/visuospatial | Total BVMT score: above first quintile of distribution = 0; lowest quintile = 1 |
| 6. TMT | TMT | Cognitive/working memory | TMT-A time ≤ 78s = 0; > 78s = 1 |
| 7. SDMT | SDMT | Cognitive/information processing | Raw SDMT score: above first quintile of distribution = 0; lowest quintile = 1 |
| 8. Have you had troubles with memory in the past month? | MSQOL-54 | Cognitive/general memory | All of the time = 1; Most of the time = 0.8; A good bit of the time = 0.6; Some of the time = 0.4; A little = 0.2; None of the time = 0 |
| 9. Difficulty concentrating or thinking in the past month? | MSQOL-54 | Cognitive/attentional control | All of the time = 1; Most of the time = 0.8; A good bit of the time = 0.6; Some of the time = 0.4; A little = 0.2; None of the time = 0 |
| 10. Been less motivated to participate in social activities in the past month? | MFIS | Psychosocial/participation | Always = 1; Often = 0.75; Sometimes = 0.5; Rarely = 0.25; Never = 0 |
| 11. Have you been happy in the past month? | MSQOL-54 | Psychosocial/mood | All of the time = 0; Most of the time = 0.2; A good bit of the time = 0.4; Some of the time = 0.6; A little = 0.8; None of the time = 1 |
| 12. Have you felt downhearted and blue in the past month? | MSQOL-54 | Psychosocial/mood | All of the time = 1; Most of the time = 0.8; A good bit of the time = 0.6; Some of the time = 0.4; A little = 0.2; None of the time = 0 |
| 13. Accomplished less than you would like as a result of emotional problems in the past month? | MSQOL-54 | Psychosocial/mood | Yes = 1; No = 0 |
| 14. Do you take more than 3 medications per day? | Medication list | Comorbidity/polypharmacy | Yes = 1, No = 0 |
| 15. Body mass index | Patient characteristics | Comorbidity/body weight | <18.5, ≥30 = 1; >25, <30 = 0.5; ≥18.5, ≤25 = 0 |
| 16. How much bodily pain have you had in the past month? | MSQOL-54 | Comorbidity/pain | Very severe = 1; Severe = 0.8; Moderate = 0.6; Mild = 0.4; Very mild = 0.2; None = 0 |
| 17. Satisfied with sexual function in the past month? | MSQOL-54 | Comorbidity/sexual | Very dissatisfied = 1; Somewhat dissatisfied = 0.5; Neither dissatisfied nor satisfied = 0 |
| 18. Difficulty getting/keeping erection (male) or inadequate lubrication (female) in the past month? | MSQOL-54 | Comorbidity/sexual | Very much a problem = 1; Somewhat of a problem = 0.5; Little of a problem = 0 |
| 19. Cardiovascular disease | Patient characteristics | Comorbidity/disease | Yes = 1; Suspected = 0.5; No = 0 |
| 20. Stroke | Patient characteristics | Comorbidity/disease | Yes = 1; Suspected = 0.5; No = 0 |
| 21. Diabetes | Patient characteristics | Comorbidity/disease | Yes = 1; Suspected = 0.5; No = 0 |
| 22. Cancer | Patient characteristics | Comorbidity/disease | Yes = 1; Suspected = 0.5; No = 0 |
| 23. Arthritis | Patient characteristics | Comorbidity/disease | Yes = 1; Suspected = 0.5; No = 0 |
| 24. Chronic lung disease | Patient characteristics | Comorbidity/disease | Yes = 1; Suspected = 0.5; No = 0 |

**Abbreviations:** MSQOL-54: 54-item multiple sclerosis quality of life questionnaire; MFIS: modified fatigue impact scale; MMSE: mini mental status examination; CVLT-II: California verbal learning test; BVMT: brief visuospatial memory test; TMT: trail making test; SDMT: symbol digit modalities test.
